# A Study of Differential Expression of Testicular Genes in Various Reproductive Phases of *Hemidactylus flaviviridis* (Wall Lizard) to Derive Their Association with Onset of Spermatogenesis and Its Relevance to Mammals

**DOI:** 10.1371/journal.pone.0151150

**Published:** 2016-03-10

**Authors:** Hironmoy Sarkar, Satyapal Arya, Umesh Rai, Subeer S. Majumdar

**Affiliations:** 1 Department of Zoology, University of Delhi, Delhi, 110007, India; 2 Cellular Endocrinology Laboratory, National Institute of Immunology, New Delhi, 110067, India; University of Hyderabad, INDIA

## Abstract

Testis of *Hemidactylus flaviviridis*, commonly known as Indian wall lizard, displays a lack of cellular and metabolic activity in regressed phase of testis during non-breeding season of the year. Retracted Sertoli cells (Sc), fibroid myoid cells and pre-meiotic resting spermatogonia are observed in such testis. This situation is akin to certain forms of infertility in men where hormone supplementation fails to generate sperm despite the presence of Sc and germ cells (Gc) in testis. In testis of lizard, spermatogenesis is reinitiated upon increased level of hormones during appropriate season (phase of recrudescence). Study of genes associated with generation of sperm, from regressed adult testis in lizard, may provide valuable information for understanding certain forms of male idiopathic infertility. Subtractive hybridization using testicular RNA obtained from the regressed and active phases of lizard reproductive cycle led to identify eight partial mRNA sequences that showed sequence homology with mice genes. We further evaluated the gene expression prolife by real-time PCR in three different reproductive phases of *H*. *flaviviridis*: regressed (pre-meiotic), recrudescent (meiotic) and active (post meiotic), for comparison with the corresponding testicular phases found in testis of 5 days (pre-meiotic), 20 days (meiotic) and 60 days (post-meiotic) old mouse. This is the first report where genes associated with progression of spermatogenesis during active phase, which follows a regressed state of adult testis, were identified in lizard and found to be conserved in mouse. Six important genes, *Hk1*, *Nme5*, *Akap4*, *Arih1*, *Rassf7* and *Tubb4b* were found to be strictly associated with active spermatogenesis in both mouse and lizard. Factors interfering with the expression of any of these genes may potentially abrogate the process of spermatogenesis leading to infertility. Such information may shed light on unknown causes of idiopathic male infertility.

## Introduction

Spermatogenesis is the complex biological process by which sperm are produced by successive mitosis and meiosis. This requires extensive intercellular coordination within the testis among different cell types, e.g., germ cells, Sertoli cells, Leydig cells, peritubular cells, etc. Intracellular coordination links to the timely expression of certain genes and their interactions, which collectively play a major role in the successful completion of sperm production.

Seminiferous tubules in the testis are surrounded by lymphatic endothelium [1]. The peritubular myoid cells and basal lamina provide structural support to seminiferous tubules within which Sertoli cell and germ cells resides. The Sertoli cells give structural framework for the organization of tubules and provide niche for the germ cell division and differentiation [2,3]. Rodents, mice and rats are most commonly used to study regulation of male fertility. In them, undifferentiated spermatogonial stem cells are situated at basement membrane either as single cell (As) or as pairs of cells (Apr) or as chains of aligned (Aal) cells [4]. Aal cells divide and differentiate into spermatocytes and after two rounds of meiosis develop into round spermatids. These round spermatids become elongated and eventually lose their large portion of cytoplasm to finally develop into sperm [5]. However, there is a remarkable variation in this process from species to species and from seasonal breeders to non-seasonal breeders.

In mouse (continuous breeder), various phases of testicular development appears as the animal matures from neonatal to adulthood. At around 5 days of post natal age (neonatal), the testis consist of undifferentiated spermatogonial cells and more than 50 percent of those remain in the resting phase with no cellular division and differentiation [6]. At 8–10 days of age, the beginning of meiosis (germ cells in leptotene stage) is apparent in a few tubules. At around 19 days, nearly half of all tubules exhibit the initiation of meiosis (presence of pachytene). A 60-day old mouse is considered an adult, with testicular sperms and reproductive maturity [6].

As opposed to mouse, most of the lizard species are seasonal breeders, they mate in spring, offspring hatches in summers. Seasonal reproduction is a tactic to make use of energy in an economical manner. The wall lizards have a prenuptial cycle of sperm maturation. Prenuptial reptiles generates sperm prior to or during the mating period [7]. *Hemidactylus flaviviridis*, commonly known as Indian wall lizard, is a seasonally breeding animal. Based on seasonal variations they have three reproductive phases within a year [8], namely- regressed (June and July), recrudescent (August and September), and active phase (October-May). Regressed Phase is the most inactive phase of spermatogenesis. There is a lack of cellular and metabolic activity in testis during this phase. Only retracted Sertoli cells and undifferentiated pre-meiotic resting spermatogonia are observed in the testis. All other advanced germ cells are absent. Peritubular myoid cells become fibroid and overall size of the testis is reduced to the smallest [9]. This is a situation akin to several forms of infertility in men [10]. In lizards, complete arrest of spermatogenesis (observed for a period of about 2 months) in regressed phase, followed by the initiation of spermatogenesis occurs through proliferation of spermatogonia in recrudescence phase. Majority of the germ cell development and maturation occurs in the active phase where sperm are released into the lumen of seminiferous tubules. In the winter months (November to January), spermatogenesis is slow and active breeding happens from February to May when sperm are continuously generated within the testis [8,9,11]. Study of genes involved in generation of sperm from regressed testis, immediately following stage of recrudescence may provide valuable information for treating infertile men having inactive spermatogenesis in spite of presence of Sc and Gc in the testis (similar to the testis of regressed phase in lizards).

We have previously reported the differential gene expression in testis of three different reproductive phases of wall lizard by microarray analysis [12]. As microarray was performed using mouse array chip, wall lizard specific gene sequences were not available for further studies. The aim of the present study was to identify the genes which are differentially expressed in the testis during active and regressed phase of reproductive cycle in wall lizards by subtractive hybridization technique. Presence of such genes, specifically expressed in testis during regressed, recrudescent and active phases of wall lizards were compared with the testis from neonatal (no spermatogenesis), pubertal (initiation of spermatogenesis) and adult phases (all stages of spermatogenesis) of mice, respectively. Such study will not only enrich our current understanding about genes involved in the onset of spermatogenesis and testicular development which are conserved across the species but will also help in addressing issues related to certain forms of infertility.

## Materials and Methods

### Tissue collection and RNA isolation

Laboratory mouse strain FVB/J was kept in animal house of National Institute of Immunology according to guidelines provided by The Committee for the Purpose of Control and Supervision of Experiments on Animals (CPCSEA), government of India. Indian wall lizards *Hemidactylus flaviviridis* were procured from a local supplier in Delhi, India, and were kept in the animal house of Department of Zoology, University of Delhi, and maintained as described earlier by us previously [13]. Adult male wall lizards, of body weight around 8 to10 grams in the months of June (during regressed phase), September (recrudescent phase) and February (active phase) were procured and maintained (12 hr light: 12hr dark) in wooden cages with wire meshes on the side and on top, and were fed live insects as food. The animals were acclimatized to the laboratory conditions under confinement for a week prior to the commencement of experiments. All the experiments regarding animals for this study were performed according to the guidelines provided and approved by Institutional Animal Ethics Committee (IAEC), University of Delhi and National institute of Immunology under guidance of The Committee for the Purpose of Control and Supervision of Experiments on Animals (CPCSEA), government of India. Isolation of testes for all three phases (active, recrudescent and regressed) was done according to a previously mentioned protocol by us [12]. For a single biological sample of testicular tissue from wall lizards, 10 to 15 from regressed, 4 to 5 from recrudescence and 2–3 males from active phase were sacrificed by decapitation. For the isolation of mouse testicular tissue from 5, 20 and 60 days post natal; the number of animals were sacrificed by cervical dislocation was 10–12, 4–6 and 2–3, respectively for each group. Testes were snap-frozen in liquid nitrogen and crushed to powder with sterile mortar and pestle. The powder were mixed in 1ml Tri reagents (Sigma-Aldrich,USA). This generated one biological replicate for each phase and, likewise, three such biological replicates were generated for each sample group. RNA isolation from tissue in Tri-reagent was done according to manufacturer’s recommendation (Sigma-Aldrich,USA). To analyze for any changes in testicular weight, tubule diameter and body weight during different testicular phases in lizards and mice, we have randomly taken three animals (six testes) from each phase in lizard and mice. Body and testes weight were recorded. All testes were used for preparing histological sections for measurement of tubule diameter.

### Histology of testis

Testes isolated from various phases of the reproductive cycle of wall lizards and different postnatal days of mice were fixed using 10 percent neutral buffered formalin, pH 7. Paraffin blocks were prepared, 5 μm sections were made on microtome machine (2040AUTOCUT, Reichert-Jung), and hematoxylin-eosin staining was performed. After DPX mounting, the sections were observed and testicular diameters were measured under bright field upright microscope (Eclipse Ti Nikon, Japan).

### Subtractive hybridization

**Fig 1:**
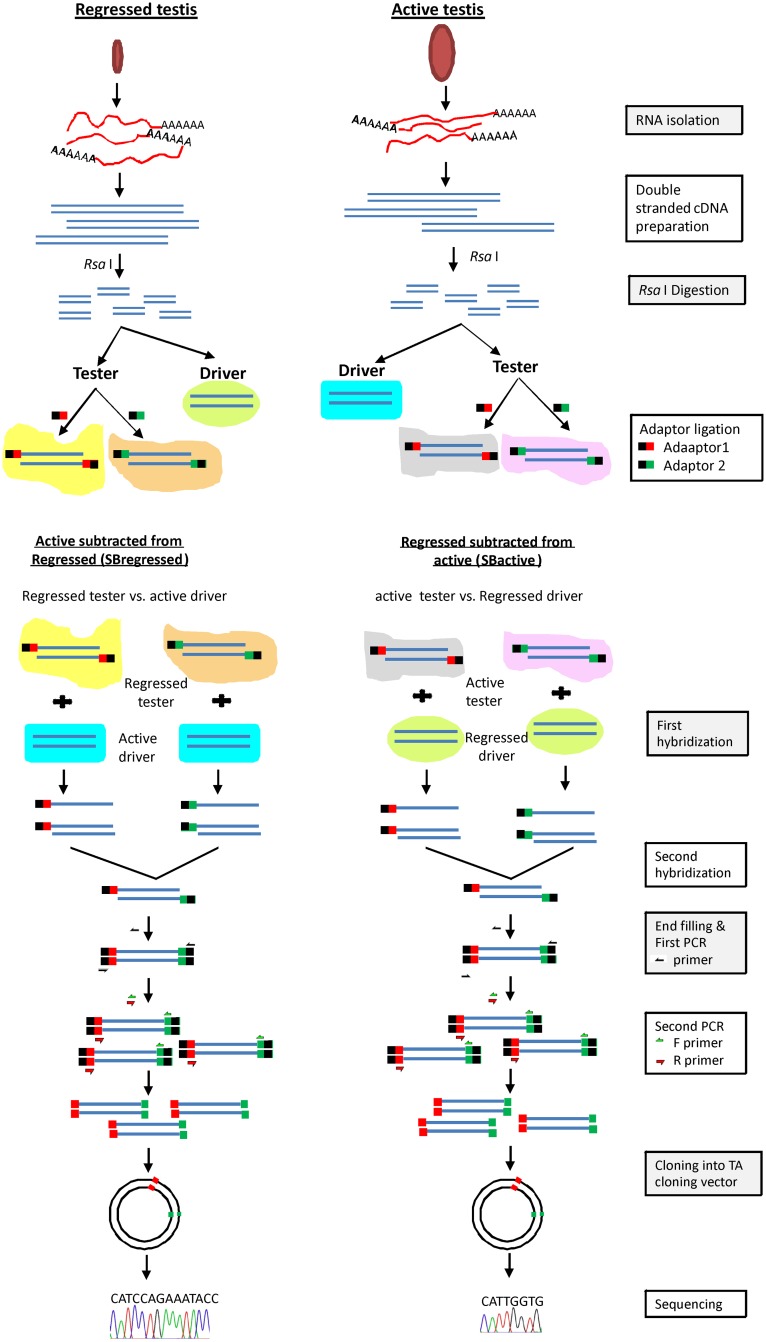
Flowchart of subtractive hybridization protocol for lizard testicular samples. Schematic representation depicting method of subtractive hybridization between RNA isolated from testes of two different reproductive phases (active and regressed phase) of wall lizard.

Subtractive hybridization was done according to the manufacturer’s protocol (Clontech,USA: cat.No– 637401; protocol no- PT1117-1). In brief, 2 μg of RNA isolated from testes in active and regressed phases were used to generate double-stranded cDNAs, which were digested with RsaI enzyme, to generate blunt–end partial cDNAs. The digested resultant of each phase was divided in two portions named tester and driver. Testers from the active and regressed phases were again divided in two halves; the one half was ligated with adaptor1 and to another adaptor2 (provided with the kit). The efficiency of adaptor ligation was determined as suggested in the kit protocol. Hybridization was performed between the active tester and the regressed driver, where regressed is subtracted from active (hereafter will be referred to SBactive) and the regressed tester and the active driver, where active is subtracted from regressed (hereafter will be referred to SBregressed). The hybridized samples were used for primary and secondary subtraction PCRs with primers provided with the kit. Subtraction efficiency was evaluated between the subtracted and un-subtracted samples of the active phase using primers specific to *H*. *flaviviridis*. PCR amplification was terminated in different cycles (28, 33, 38 and 42) and all PCR products were run on gel to validate the subtraction efficiency. The secondary PCR products, contained partial cDNAs, were ligated to a TA cloning vector in multiple cloning site (Promega Corp, USA), and were transformed into *E*. *coli*. From each SBactive and SBregressed sample, randomly130 positive colonies were selected to perform colony-PCR to verify the insert size in TA cloning vector. Colonies showing higher molecular mass with a single amplification product in the colony-PCRs were considered; 48 different colonies, of each SBactive and SBregressed sample were selected. pDNA from those selected colonies were isolated with GenElute plasmid miniprep kit (Sigma, USA). The region of pDNA having partial cDNAs were sequenced (Amnion Biosciences, India). A flowchart regarding the above-described subtractive hybridization process is illustrated in Fig 1.

### Sequence analysis and phylogenetic tree preparation

To find the identity of the partial cDNAs, the Basic Local Alignment Search Tool (BLAST) was used to align with nucleotide sequences deposited in the NCBI databases. Identities of partial cDNAs were assigned from the best aligned sequence having highest identity score and query coverage with least E value.

A phylogenetic tree was constructed with the partial cDNAs and their corresponding ortholog sequences in other species. MEGA5 software was used for generation of the phylogenetic tree by neighbor-joining statistical method and the bootstrap phylogenetic tree construction method, having a bootstrap replication of 1000 [14].

### Reverse Transcription and Real-Time PCR

1 μg of RNA, isolated from the testes samples of lizards and mice, was treated with DNaseI according to the manufacturer’s protocol (Thermo Scientific, USA). To generate cDNAs form the treated RNAs, M-MLV reverse transcriptase (Promega Corp, USA) were used with oligo (dT)_15_ as primer in a final volume of 25μl. cDNA was diluted 3 times with nuclease-free water, and subsequent PCRs were carried out using 1μl of the diluted cDNA as template.

Gene-specific oligo-primers for real-time PCR were designed using software tools: PrimerBlast (http://www.ncbi.nlm.nih.gov/tools/primer-blast/ as on 22 July, 2015) and GeneRunner (http://generunner.net/ as on 22 July, 2015). Reference sequences for gene-specific primers were acquired from NCBI database for mice and partial cDNA sequences, obtained from subtractive hybridization, for wall lizards. Primer sequences are listed in Table 1.

**Table 1:** List of primers for real-time PCR.

Gene ID	Species	Primer sequences (5'-3')		Product length	NCBI Acc No
		Forward	Reverse		
*Hk1*	Lizard	GAATGACACGGTGGGAGTC	TGTATGCCTCCTATGTTCTC	126	JZ822542
	Mouse	CAGTGTGAAGTCGGCCTGAT	GGACCCATCATCCCCAAAGG	141	NM_001146100.1
*Nme5*	Lizard	GGGATTATGTTCAATTAGCC	CTATTCCTCAGCTGAAAGAG	199	JZ822546
	Mouse	CAGGTTCATGTTTCCAGCCG	TCCTTGAAGTAGGGTTGGCG	100	NM_080637.3
*Akap4*	Lizard	TGGAAAGTTGAGAGAGGAGG	GGAAGTGGAACTGTACCTAG	105	JZ822547
	Mouse	AGGACAACAAGATCAGGACCG	CAGCAGCACCCTTGGAATC	89	NM_001042542.2
*Bco2*	Lizard	ACTATACAAAGCCCCTCATG	GCTGCAGTAGGAATAGGTC	120	JZ822548
	Mouse	ATGTTGGGACCGAAGCAGAG	GCAACGCCATTCCATCAAACC	196	NM_133217.3
*Arih1*	Lizard	ATGTCTTGCAGCGAATCTTG	AACCAGTCCATTATCTTCG	107	JZ822545
	Mouse	CAGTGTCGTGCCACACTCAT	CGAAAGCACCTCTGTGGCAT	120	NM_019927.2
*Rassf7*	Lizard	GGCTAGCCCTGACAAACGT	AGCCAAGTGTGGACAGTATG	128	JZ822549
	Mouse	ACAGGTCGATTTGTCCTTGT	GTATCGCCGATGGCTTTGG	229	NM_025886.3
*Hmgb1*	Lizard	GTAGGGTATGCAGAACGAAG	GACTTGTCCTGTGTTATACC	100	JZ822550
	Mouse	GGACTCTCCTTTAACCGCCA	CCTTCGCTGGGACTAAGGTC	148	NM_010439.3
*Tubb4b*	Lizard	TACAGCTGAGGAGGAAGGAG	GATGACACCAGGAAGAAAGG	170	JZ822551
	Mouse	CACTTACCACGGAGATAGCGA	ACCTTCTGTGTAGTGCCCCTT	229	NM_146116.2
*Gapdh*	Lizard	ACACAGTCCATGCCATCACA	GACCTTGCCAACAGCCTTA	134	JF303078.1
*Ppia*	Mouse	GTCGCTTTTCGCCGCTTGC	TCTGCTGTCTTTGGAACT	127	NM_008907.1

Real-time PCR was carried out with syber green using Mesa-green master mix kit (Eurogentech, Belgium) in Eppendorf Realplex mastercycler machine (Eppendorf, Germany). Reaction setup and PCR primer efficiency calculations were done by previously described procedure by us [15,16]. Primers with an efficiency of 1±0.2 were considered for further use. To evaluate the gene expression pattern in the testis of three reproductive phases of wall lizards and the three developmental phases of mice, three biological replicates were run for every phase of testicular sample, and for each biological sample, there were two technical replicates along with one control for reverse-transcriptase and one non-template control. The expression of *Gapdh* (Glyceraldehyde-3-Phosphate Dehydrogenase) for wall lizard and *Ppia* (Peptidylprolyl Isomerase A) for mice was used as reference gene for normalizing the target genes expression. Calculation for relative fold change with respect to the active phase in wall lizards and 60 days in mice was performed by 2^-ΔΔct^ method as previously described by us [17].

A clustered image heat-map was generated with the relative fold change values, using online tool CIMminer (http://discover.nci.nih.gov/cimminer/home.do as on date 22 July, 2015) to represent the gene expression profile between wall lizards and mice contemporary testis. For the generation of heat map methods like euclidean, average linkage, and quantile were used as distance method, cluster algorithm, and binning method, respectively.

### Correlation between testis weight and gene expression

Testes weights (mg) of nine testicular samples from wall lizards (three biological replicates for each of three phases) were assigned percentage values by considering the highest value amongst all of them as 100. Here, the highest value came from one biological replicate of the active phase of wall lizard. In a similar manner, mice testicular weights from nine testicular samples of mouse (three biological replicates for each three developmental phases) were assigned percentage values by considering the highest value amongst all of them as 100. A graph was generated for each gene, where y axis was assigned to the relative fold change values (obtained by 2^-ΔΔct^ method) of testicular samples against their corresponding testis-weight in percentage on x axis. A linear regression line was drawn by connecting the expression values plotted on the graph. In the graph, independent linear regression line was generated for wall lizards and mice.

### Statistical analysis

The relative fold change in gene expression data were represented as ±SEM (n = 3). Statistically significant difference among the samples were analyzed by one-way analyses of variance (ANOVA) followed by Newman-Keuls multiple comparison test. Difference in gene expression was considered significant when the p value was found to be less than 0.05. Correlations were determined between testis wt. and relative gene expression by Pearson's correlation coefficient analysis. p<0.05 was considered statistically significant correlation. All statistics and graph preparation were done by GraphPad prism software (USA) version 5.0.

## Results

### Comparison of seasonal testicular growth in wall lizards with the testicular development in mice

**Fig 2:**
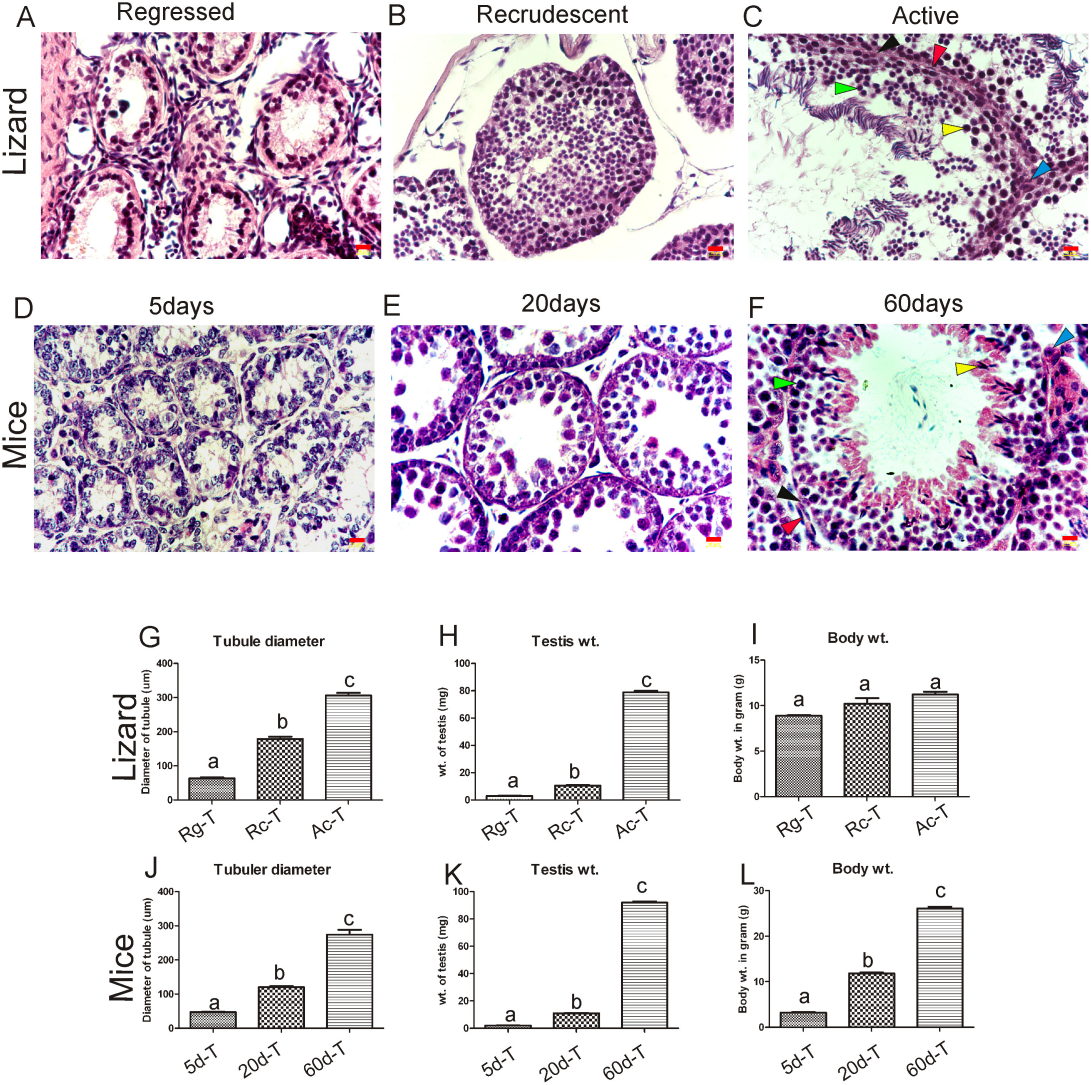
Comparative analysis of testicular development in wall lizard and mice at different reproductive phase of testes. (A), (B) and (C) represent the cross section of testis isolated from regressed, recrudescent and active phase of reproductive cycle in wall lizard, respectively. Figure (D), (E) and (F) represent testis isolated from 5 day, 20 day and 60 day old mouse respectively. In the figure (C & F), as for representation, Leydig cell (blue), peritubular cell (red), Sertoli cell (black), germ cell (green), and spermatids (yellow) are shown with respective arrowhead. Figure (G) & (J) shows comparison of tubular diameter (n = 6), (H) & (K) shows comparison of testis weight (n = 6), (I) & (L) depicts the comparative analysis body weight (n = 3) between wall lizard and mice, respectively. One way analysis of variance followed by newman-keuls multiple comparison test was performed to get P value as significance level p<0.05. In a graph, different letters above the bars denotes significant difference in values (p<0.05), Bars refers to S.D. between replicates (n = 3). Scale bar on the figure A-F indicates 0.01mm. Rg-T, Rc-T, Ac-T are testes isolated from regressed, recrudescent, and active phase of wall lizard, respectively. 5d-T, 20d-T, and 60d-Tdenotes testes isolated from 5 days, 20 days and 60 days old mouse, respectively.

Cross section of testis from the regressed phase of a wall lizard showed small seminiferous tubules having small lumen, consisting mostly Sertoli cells and very few early germ cells (Fig 2A). The 5-day old mouse testis (Fig 2D) also had similar structure having mostly Sertoli cells and few early germ cells, except that it did not have any lumen in the seminiferous tubule. Fig 2B and 2E show cross sections of the recrudescent phase of wall lizards and 20-day old mouse testis, respectively. Testis from both species consisted of mostly pre-meiotic and meiotic stages of germ cells. Testis from active phase of wall lizards (Fig 2C) and adult 60-day old mice (Fig 2F) showed presence of all stages of germ cells along with spermatids. In the both testis, tubular diameter and testicular weight increased (Fig 2G, 2H, 2J and 2K) from regressed to active (in wall lizards) and from 5 days towards 60 days testis (in mice). The Body weight of wall lizards did not differ significantly among various reproductive phases (Fig 2I); whereas, body weight of mouse increased with age (Fig 2L).

### Subtractive hybridization between active and regressed phase of testicular RNA from wall lizards

Subtractive hybridization was done to identify genes that are differentially expressed between active and regressed phases of the testis. To get transcripts that were highly expressed in the active phase in comparison to the regressed phase, the testicular RNA from regressed phase was subtracted from the testicular RNA of active phase (SBactive). For identification of transcripts expressed more in the regressed phase compared with the active phase, the active phase testicular RNA was subtracted from the testicular RNA of regressed phase (SBregressed).

**Fig 3:**
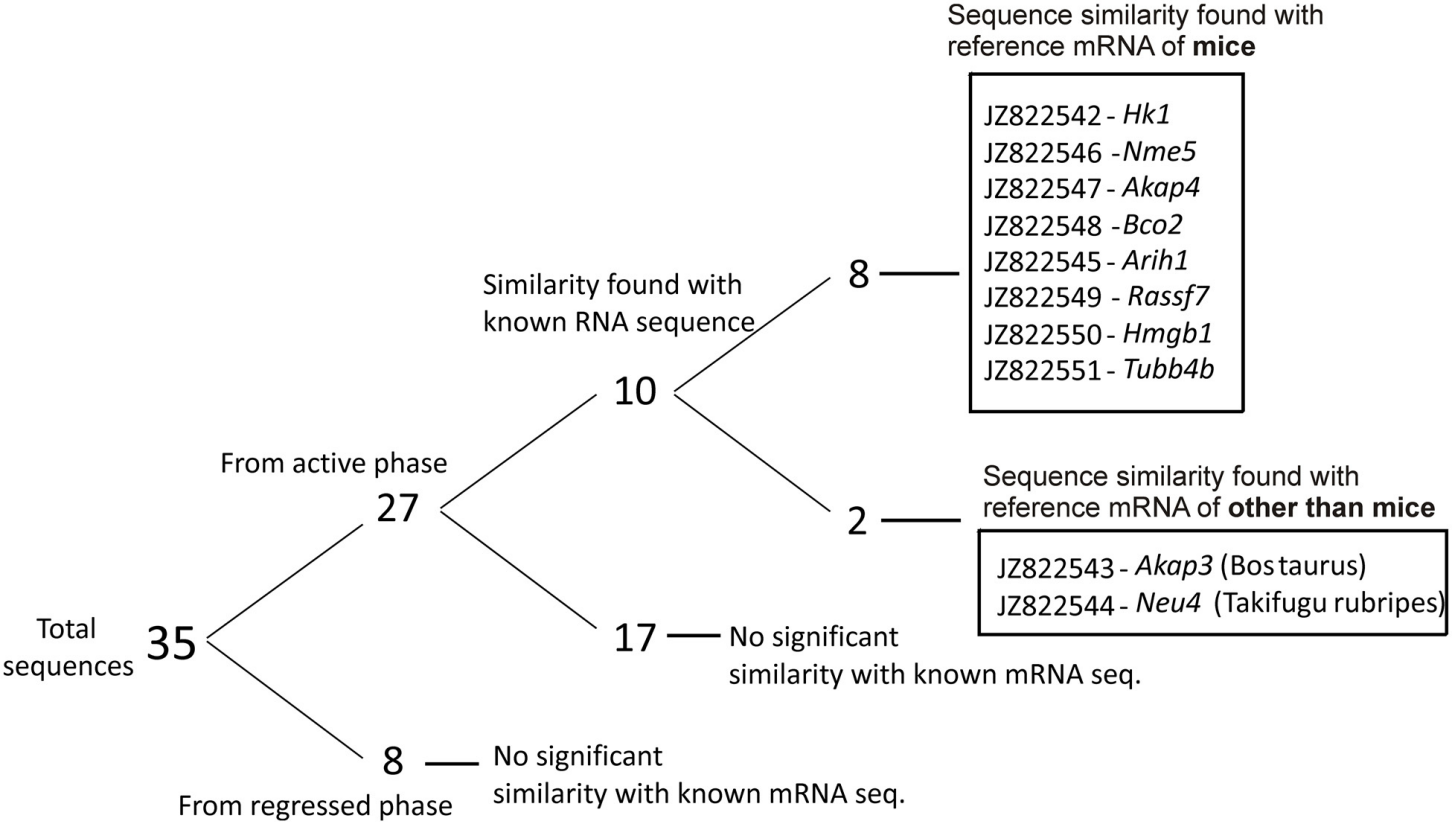
Summarized result of subtractive hybridization analysis. Distribution of total partial sequences, obtained from subtractive hybridizations analysis between testicular RNA isolated from regressed and active phase of wall lizard.

Sequencing of the fragments of transcripts, obtained from SBactive and SBregressed provided 35 unique sequences (NCBI Acc No- JZ822542 –JZ822576). Out of these, 27 sequences were from the active phase (SBactive) and 8 were from the regressed phase (SBregressed). Out of 27 sequences in the SBactive sample, 10 sequences were found to have similarity with known gene sequences from different species (Fig 3). Out of these 10, 8 partial gene sequences (*Hk1*, *Nme5*, *Akap4*, *Tubb4b*, *Arih1*, *Rassf7*, *Hmgb1* and *Bco2*) of the wall lizards were found to have high homology with ortholog sequences in mice (Table 2). These 8 genes were from SBactive group and were used for further studies.

**Table 2:** List of partial transcript sequences of lizard and their sequence similarity with homolog-genes of mice.

GenBank Acc No	Length (bp)	BLAST result in NCBI database			Gene	Gene symbol
		Acc No	E-value	Identity		
JZ822542	281	NM_001146100.1	7.E-07	0.67	Hexokinase 1 (Hk1)	*Hk1*
JZ822546	209	NM_080637.3	5E-26	0.75	NME/NM23 family member 5	*Nme5*
JZ822547	665	NM_001042542.1	2E-32	0.69	A kinase (PRKA) anchor protein 4	*Akap4*
JZ822548	120	NM_133217.3	5E-10	0.76	Beta-carotene oxygenase 2	*Bco2*
JZ822545	154	NM_019927.2	2E-48	0.88	Ariadne ubiquitin-conjugating enzyme E2 binding protein homolog 1	*Arih1*
JZ822549	486	NM_025886.3	6E-36	0.73	Ras association (RalGDS/AF-6) domain family (N-terminal) member 7	*Rassf7*
JZ822550	420	NM_010439.3	1E-24	0.77	High mobility group box 1	*Hmgb1*
JZ822551	193	NM_146116.2	2E-06	0.82	Tubulin, beta 4B class IVB	*Tubb4b*

A phylogenetic tree was constructed using the bootstarp method, with known homolog sequences found in NCBI databases (S1 Fig). The sequence of *Arih1* (JZ822545) from *H*. *flaviviridis* was found to be nearest to *Anolis* with the highest homology, and formed an altogether different node. The sequences of *Nme5* (JZ822546) and *Tubb4b* (JZ822551) were in greater homology with *Z*. *albicollis*. Sequences *Bco2* (JZ822548), *Rassf7* (JZ822549) and*Hmgb1* (JZ822550) were in greater homology with *Xenopus*. The sequence similarities of *Hk1* (JZ822542) and *Akap4* (JZ822547) found to be higher with *Drosophila*.

### Comparative gene expression in the testis of wall lizards and mice by real-time PCR analysis

**Fig 4:**
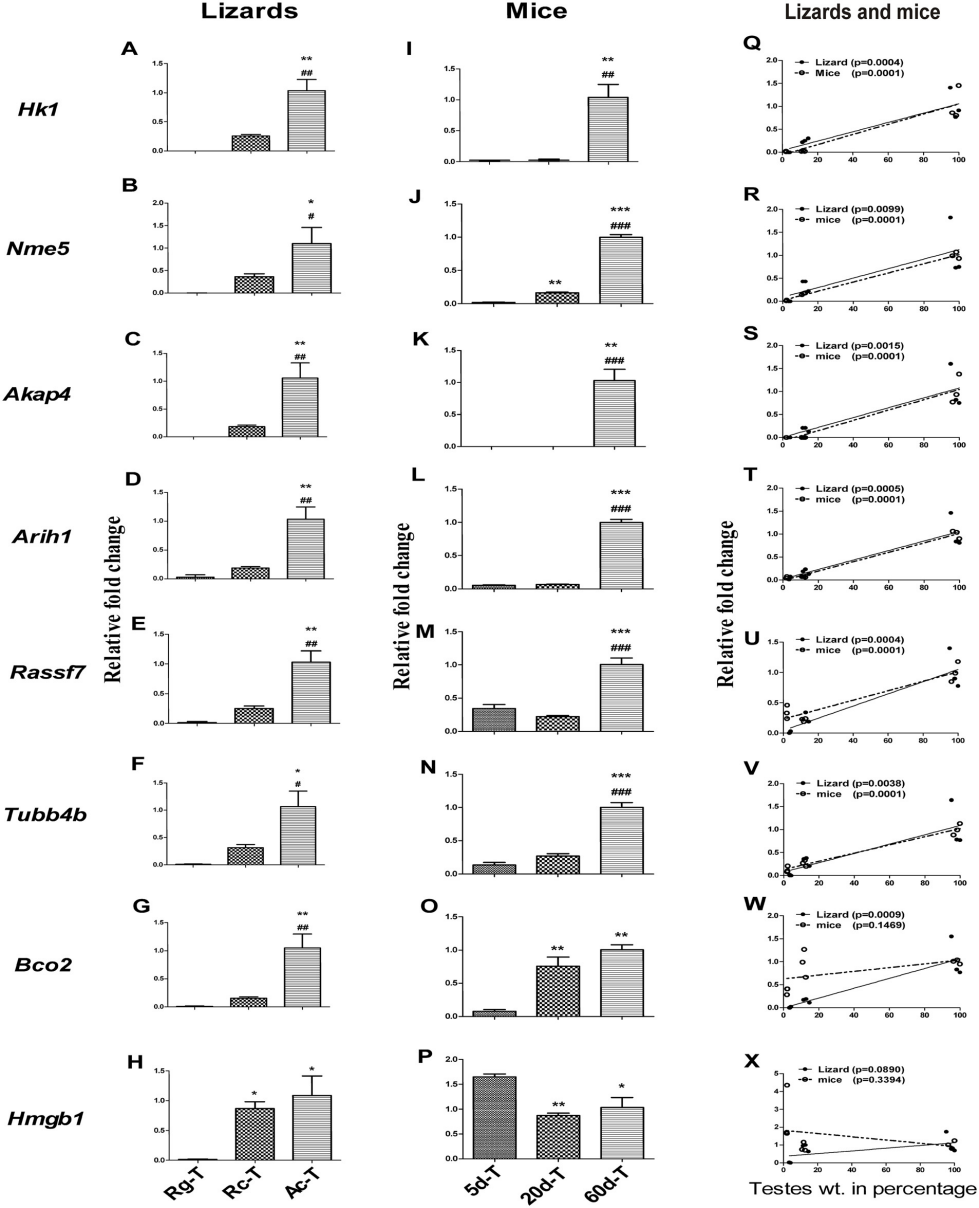
Comparative analysis of mRNA expression of selected genes in testis of wall lizards and mice. Shows level of mRNA expression of *Hk1* (A& I), *Nme5* (B& J), *Akap4* (C& K), *Arih1* (D &L), *Rassf7* (E & M), *Tubb4b* (F & N), *Bco2* (G & O) and *Hmgb1* (H & P) in three reproductive phases of wall lizard and three developmental stages of mice testis respectively. Normalization of gene expression was done with reference gene *Gapdh* for wall lizard and *Ppia* for mouse. Correlation between relative fold change in genes expression and testis weight in wall lizard and mice for genes*Hk1* (Q), *Nme5* (R), *akap4* (S), *Arih1* (T), *Rassf7* (U) and *Tubb4b* (V). *Bco2* (W), and *Hmgb1* (X) were analyzed. A solid or broken line in scatter plots denotes linear regression. Open circle with dotted line represents mice and closed circle with solid line represents wall lizard relative gene expression with respect to testicular wt. in percentage. Rg-T, Rc-T, Ac-T are testes from regressed, recrudescent, and active phase of wall lizard. 5d-T, 20d-T, and 60d-T are testes from 5 days, 20 days and 60 days old mice. *p<0.05, **p<0.01 and ***p<0.001 represents the comparison of Rg-T with Rc-T and Ac-T in wall lizards and 5d-T with 20d-T and 60d-T in mice. ^#^p<0.05, ^##^p<0.01 and ^###^p<0.001 represents the comparison of Rc-T with Ac-T in wall lizards and 20d-T with 60d-T in mice.

In order to analyze the mRNA expression pattern of 8 genes *Hk1*, *Nme5*, *Akap4*, *Arih1*, *Rassf7*, *Tubb4b*, *Bco2*and *Hmgb1*in different testicular phases of wall lizard and mice, real-time PCR was performed using testicular RNA. Except *Hmgb1*, rest other 7 genes were significantly high in the active phase of testis as compared to the regressed and recrudescent phases of wall lizards’ testis (Fig 4A–4G). Significantly higher expression of six genes *Hk1*, *Nme5*, *Akap4*, *Arih1*, *Rassf7* and *Tubb4b* were found in the 60 days old mouse testes as compared to 5 days and 20 days old testis (Fig 4I–4N). Interestingly, the expression of *Nme5* and *Bco2* were significantly higher in the 20-day old testis as compared to 5-day old testis in mice (Fig 4J and 4O). In wall lizards as well as in mice, *Hk1*, *Nme5*, *Akap4*, *Arih1*, *Rassf7* and *Tubb4b* expression were found to have significant correlation with testicular weight (Fig 4Q–4V). The increase in expression of *Bco2* in 20 days old mice did not have any significant correlation with the testicular weight, although the increase in expression in the wall lizards’ testis was found to have significant positive correlation with increase in testicular weight of wall lizards (Fig 4W). The relative expression of *Hmgb1* was found to be significantly higher in the recrudescence phase compared with the regressed phase of wall lizards’ testis. Comparable level of expressions was found between the recrudescence and the active phase of the wall lizards’ testis (Fig 4H). In the mice testis, the expression level of *Hmgb1* was found to be similar in day 20 and day 60, however, there was significantly higher expression observed in the 5-days as compared to that at 20-day and 60-day testes (Fig 4P). Neither in wall lizards nor in mice, was the expression level of *Hmgb1* found to be correlated with the increased testicular weight (Fig 4X).

**Fig 5:**
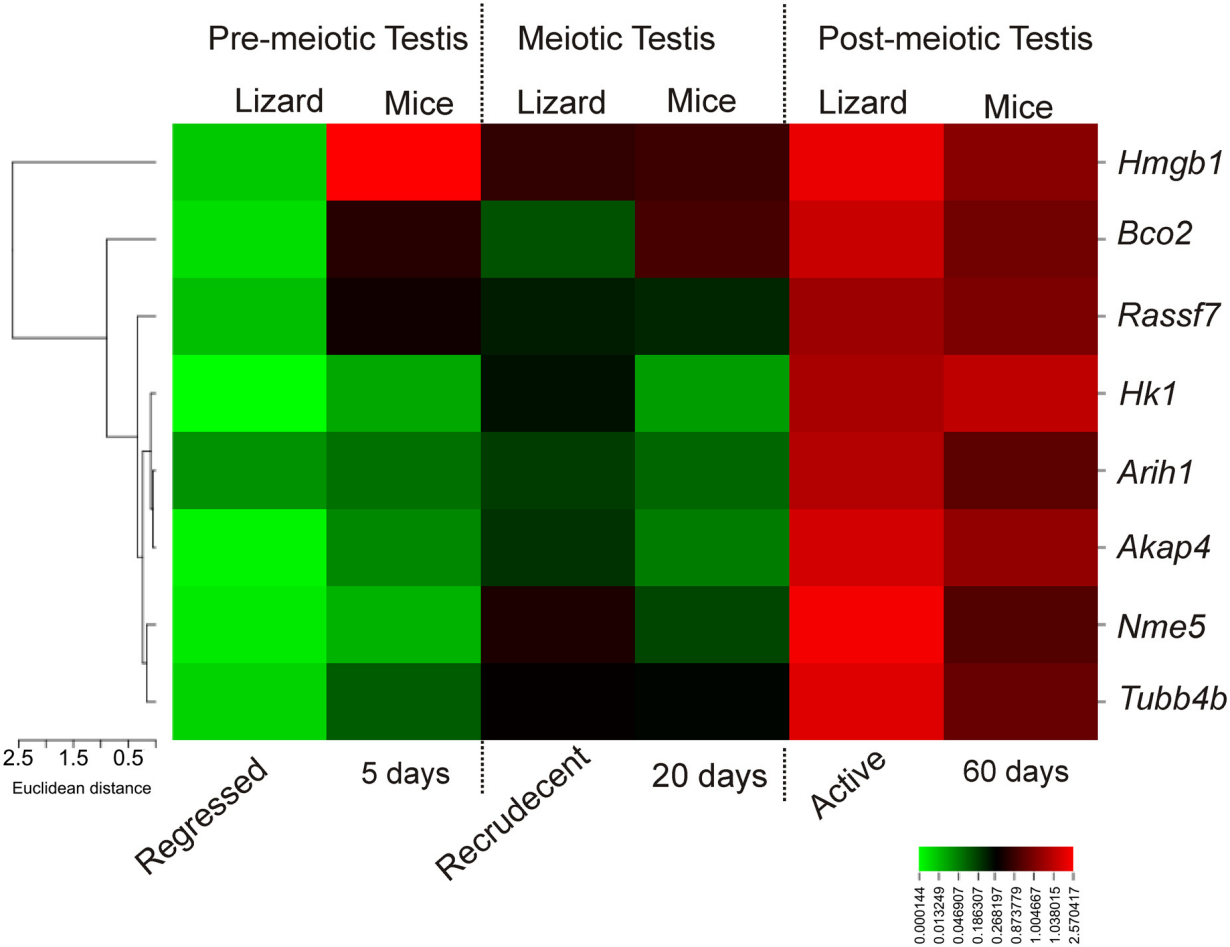
Heat map analysis of gene expression between lizards and mice. Heat map analysis showing differential gene expression pattern in pre-meiotic, meiotic and post-meiotic testes of wall lizard and mice. The relative fold change in gene expression for genes *Hk1*, *Nme5*, *Akap4*, *Arih1*, *Tubb4b*, *Rassf7*, *Bco2*, and *Hmgb1* were compared in corresponding testes of wall lizards and mice. Color from red to green indicates high to low expression.

Relative fold change in gene expression values of all 8 genes were used to generate a heat map to compare the expression status between wall lizards and mice on similar testicular phases like pre-meiotic, meiotic and post-meiotic (Fig 5). The expression of *Hmgb1* was found to be high in pre-meiotic testis of mice, but not in the testis of wall lizards. The expression of *Hmgb1* in meiotic and post-meiotic testis in wall lizards and mice were found to be comparable. We found that *Hk1*, *Nme5*, *Akap4*, *Arih1*, *Rassf7*, *and Tubb4b and Bco2* had a similar high expression in the testis of wall lizards during active phase and at 60days in the mice. *Hk1*, *Arih1*, *Akap4* and *Nme5* had comparable expressions in the regressed phase in wall lizards and 5 days old mice testis. *Bco2*, *Rassf7* and *Tubb4b* had higher expression in testis of 5 days old mice compared to testis in regressed phase in the wall lizard. *Rassf7*, *Aih1* and *Tubb4b* had a comparable expression in the testis of recrudescent phase of wall lizard and in 20-day old mice testis. The expression of *Bco2* was found to be high in testis of 20days old mice than in the testis of recrudescent phase of the wall lizard. During recrudescent phase of the wall lizard, expression status of *Hk1* and *Akap4* were higher as compared to the testis of 20 days old mice. There was higher abundance of *Nme5* in the testis from recrudescent phase of lizards although it was not in testis of 20 days old mice (Fig 5) which is a comparable testicular phase with recrudescent of lizards.

### Probable protein-protein interactions of differentially expression genes in testicular phases of wall lizards and mice

**Fig 6:**
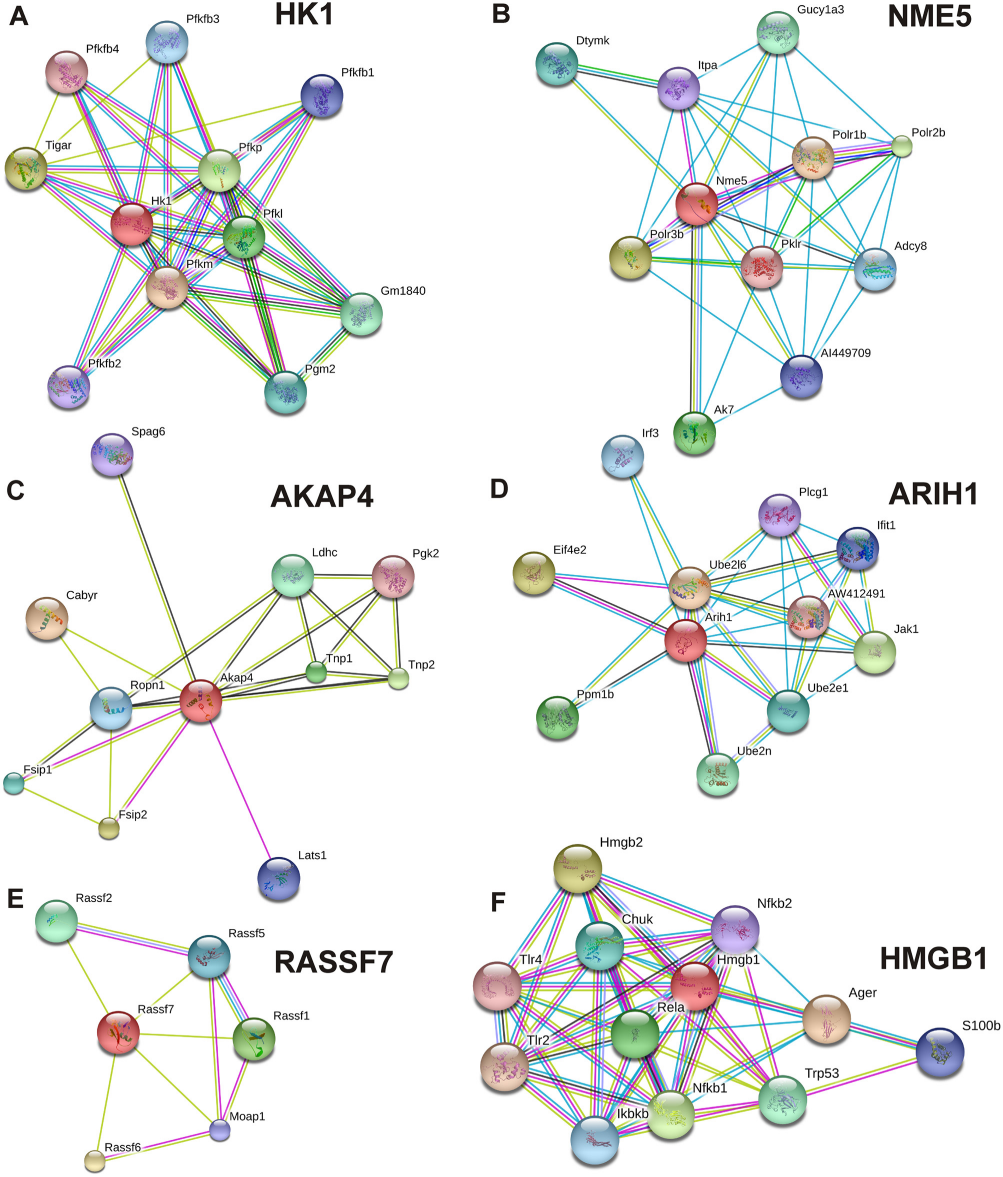
Predicted interaction among proteins. Probable interactome of different proteins, of which mRNA were differentially expressed in different testicular phase of wall lizard mice. Probable interacting partner of gene products, *Hk1* (A), *Nme5* (B), *Akap4*(C), *Arih1* (D), *Rassf7* (E), and *Hmgb1* (F) with other proteins as per STRING database.

In the STRING database, there were known and predicted protein-protein interactions of *Hk1*, *Nme5*, *Akap4*, *Arih1*, *Rassf7*, and *Hmgb1* (Fig 6). HK1 interacted with PFKM, TIGAR, PFKP, PFKL, PGM2, PFKFB3, PFKFB1, PFKFB2, and PFKFB4. NME5 interacted with POLR1B, POLR3B, POLR2B, AK7, GUCY1A3, DTYMK, ADCY8, ITPA, and PKLR. AKAP4 interacted with CABYR, FSIP2, TNP2, TNP1, LDHC, FSIP1, ROPN1, LATS1, SPAG6, and PGK2. ARIH1 interacted with UBE2L6, EIF4E2, JAK1, PPM1B, UBE2N, UBE2E1, IRF3, IFIT1, and PLCG1. RASSF7 interacted with RASSF6, RASSF1, RASSF2, RASSF5, and MOAP1. HMGB1 interacted with AGER, HMGB2, NFKB1, RELA, TRP53, CHUK, IKBKB, S100B, NFKB2, TLR4, and TLR2. The interaction score and name of interacting proteins are mentioned in S1 Table. Interactome of *Bco2* and *Tubb4b* were not found in the STRING database.

## Discussion

In this study, we have compared the gene expression profiles in the testis of wall lizards and mice during various phases of testicular activity (pre-meiotic, meiotic and post-meiotic). Earlier we have compared the differential transcriptome of three reproductive phases of testis from wall lizard [12]. Since lizard array chip was not available, wall lizard testicular RNA was hybridized on known mouse array chip. As gene sequences of wall lizard and mouse vary widely, the expression signals due to hybridization was low in confidence. Additionally as lizards gene sequences are not available, the validation of microarray data and further expression studies with gene specific primers are not efficient. To overcome these problems, we have compared the testicular RNA expression profile between regressed and active phase of spermatogenesis in lizard by subtractive hybridization.

Thirty five unique partial RNA sequences were identified in testis of wall lizard. The 17 and 8 partial sequences from active and regressed phase, respectively, showed no significant similarity with known mRNA sequences in the database. The reason for the lack of similarity of these partial sequences with known mRNA sequences may reside in the lack of longer sequence reads (eg. JZ822572 and JZ822543), which is necessary to attain a significant match. There were other sequences, which were found to be relatively longer and showed partial similarity to short region of more than one mRNAs (eg. JZ822573, JZ822574, JZ822575); the query coverage for these sequence alignments were also very low (below 40%). Therefore, these 17 and 8 sequences could not be reliably identified as mRNA sequences of known genes. Hence, in the present study we could not consider these partial sequences for further experiments.

Ten partial RNAs among 35, showed sequence homology with mRNA sequences of orthologus genes from other species and found to be abundant in the testis during active phase compared to regressed phase of wall lizards. Out of 10 sequences, 8 showed sequence similarity with corresponding mice gene sequences. In order to evaluate testicular phase related similarity in the expression pattern between two species, we analyzed the expression profiles of these 8 genes using real-time PCR in different phases of mice testicular development and lizard reproductive cycle. 7 genes, *Hk1*, *Nme5*, *Akap4*, *Arih1*, *Rassf7*, *Bco2* and *Tubb4b* were found to be uniformly associated with testis with active spermatogenesis in both the species. Expression of 6 genes *Hk1*, *Nme5*, *Akap4*, *Arih1*, *Rassf7* and *Tubb4b* showed positive correlations with increased testicular weight in both wall lizards and mice, that represents their conserved pattern of expression between these two species. Specifically higher expression of *Hmgb1* in testis of 5 days old mice suggested that the regressed testis cannot be considered as neonatal testis in terms of developmental status.

**HK1** is an important enzyme for glucose metabolism. HK1 localizes on the outer membrane of mitochondria [18] and facilitates the conversion of glucose to glucose-6-phosphate. The isoform of HK1, spermatogenic cell-specific type 1 hexokinase (HK1S) was found in the germ cells and abundantly in sperm and located mainly in the principal piece of the sperm flagellum to help in sperm motility [19,20]. As glucose metabolism is very important in cellular processes, our finding of a high expression of *Hk1* in the spermatogenically active testis of wall lizards and mice suggested its requirement for spermatogenic maintenance. Interaction of HK1 with glucose metabolism associated proteins (PFKM, PFKP, PFKL and PGM2) as well as fructose metabolism associated proteins (TIGER, PFKFB1, PFKFB2, PFKFB3 and PFKFB4) based on interactome database, suggested HK1 is important regulator of energy source to actively dividing spermatogonia. **NME5** is a NME/NM23 family member protein, also known as Nm23-h5. This is a nucleotide diphosphate (NDP) kinase that catalyses phosphoryl exchanges between nucleoside di- and tri-phosphates [21]. The mRNA expression was found in spermatogonia and in early spermatocytes in human and mouse testis [21,22], and the proteins were located in the sperm head and flagella [23]. In the interactome data, there are a few proteins that showed interactions with NME5, like AK7 (helps in the maintenance of ciliary structure and function), DTYMK, ITPA and PKLR. In our knowledge, the expression of Nme5 has not been reported except in humans and mice [22,23]. **AKAP4**, which is a member of the AKAP family, helps in binding to the regulatory subunit of protein kinase A (PKA), which works as a scaffold protein. AKAP4 is found in the fibrous sheath in sperm and helps in giving mechanical support to the sperm tail [24]. Targeted disruption of this gene leads to lack of sperm motility in mice, possibly due to failure of signal transduction and glycolytic enzymes to be associated with the fibrous sheath of sperms [25]. The mRNA expression of AKAP4 also found in adult testis of *Macropus eugenii*, located in the cytoplasm of round and elongated spermatids, and in the sperm tail [26]. In the interactome database, it interacted with FSIP1 and FSIP2, which are fibrous sheath interacting proteins, and with germ-cell-specific proteins like SPAG6 and PGK2, and TNP1 and TNP2, which are nucleosome compaction proteins. **BCO2** enzyme is a mitochondrial carotenoid-oxygenase, which catalyses asymmetric cleavage of carotenoids and protects the cells from carotenoid induced oxidative stress [27]. We found a higher expression of *Bco2* at 20 days (meiotic) and 60 days (post-meiotic) in comparison to that in 5 days old mice testis. This higher expression of *Bco2* in testis showing meiosis can be correlated with the requirement of retinoic acid (RA) for the meiotic initiation of germ cells. When germ cells undergo meiosis, they require retinoic acid [28] which is product of beta-carotene. Hence, there is a possibility that during meiosis, germ cells may undergo oxidative stress due to higher amount of beta-carotene; therefore, *Bco2* may create a balance for maintaining the right amount of beta-carotenoid in the germ cells. **ARIH1** can induce aggresome formation and helps in the ubiqutinilation of target proteins [29,30]. It is also found to be the marker for proliferation [31]. As the active testis of wall lizards and adult testis of mice are highly proliferative, a higher expression of *Arih1* in those phases of testis in wall lizards and mice can be justified. In the predicted interactome database, this protein interacts with the JAK1, which is a protein-tyrosine kinase found in human spermatozoa [32] and helps in germ cell differentiation in *Xenopus* [33].**RASSF7** is a ras association domain-containing protein 7, which negatively regulates stress-induced JNK activation [34]. It is also a regulator of microtubule dynamics in a cell and is required for mitosis [35]. The important paralog of this gene is*Rassf8*, found to be essential for maintaining adherent junctions (AJ) in epithelial cells and has a role in epithelial cell migration [36]. We observed a higher expression *Rassf7* in the testis of active phase and adult testis of wall lizards and mice, respectively. In the interactome data, it showed interaction with other ras-associated proteins. **TUBB4B** is a tubulin protein. We found its expression in the adult testis of mice and active testis of lizards, from which we can infer that the requirement of this component of microtubules may be higher in mature testis for providing structural support to large number of germ cells. **HMGB1** is a high-mobility group binding protein. The expression of *Hmgb1* was found to be comparable during recrudescence and active phase of lizards. This was also comparable in 20 and 60 days old testis of mice. However, this was highest in neonatal testis (5 days) of mice. Interactome data of HMGB1, suggested its interaction with several proteins related to PI3K-Akt signaling pathway, like NFKB1, RELA1, CHUK, and IKBKB, which are known to play important role in cellular growth and differentiation. The *Hmgb1* gene was found to be one of the 159 transcripts from testicular biopsy where the expression was found to be high in idiopathic infertile patients of AZFc microdeletion [37]. In many patients the cause of infertility was due to severe hypo spermatogenesis, this can be because the testis remains in state of immaturity which fails to develop sufficient functional sperm. The higher expression of *Hmgb1* in neonatal (immature) testis of mice and testis of infertile patients, led us to hypothesize that higher expression of HMGB1can keep the testis in immature state whereas moderate expression is seen during spermatogenesis.

In our knowledge this is the first report where increased expression of certain genes associated with active spermatogenesis was commonly observed in lizard and mammalian testis. The increase in six commonly identified genes during active phase was observed as compared to regressed state of the testis from lizard and neonatal testis of mice. This not only showed that these genes are conserved across the species but also indicated their strict association with spermatogenesis. Impairment in expression of any of these genes may potentially disturb the process of spermatogenesis leading to infertility. This information may be crucial in divulging unknown causes of idiopathic male infertility and hence should be studied further in detail.

## Supporting Information

S1 Fig Phylogenetic tree with partial sequences of lizards obtained from subtractive hybridization of testicular samples. Phylogenetic tree for the genes, *Hk1* (A), *Nme5* (B), *Akap4*(C), Bco2 (D), *Arih1* (E), *Rassf7* (F), *Hmgb1* (G), and*Tubb4b* (H).
(TIF)

S1 Table Predicted interacting proteins and their score of interaction-confidence. (DOC)
