# Supplementary material for: A Study of Differential Expression of Testicular Genes in Various Reproductive Phases of Hemidactylus flaviviridis (Wall Lizard) to Derive Their Association with Onset of Spermatogenesis and Its Relevance to Mammals
Source: PLoS One. 2016 Mar 10;11(3):e0151150. doi: 10.1371/journal.pone.0151150 (PMC4795608; doi:10.1371/journal.pone.0151150)
Supplement: S1 Table — (DOC) [file pone.0151150.s002.doc]

**S1 Table .** **Predicted interacting proteins and their score of interaction-confidence.** (Score 1 is the highest and 0 is the lowest, source- STRING database)

|  | Gene product | | Interacting proteins ID | | Protein name | | Score | |
| --- | --- | --- | --- | --- | --- | --- | --- | --- |
|  | HK1 | | Pfkm | | Phosphofructokinase | | 0.99 | |
|  |  | | Tigar | | RIKEN | | 0.96 | |
|  |  | | Pfkp | | Phosphofructokinase, | | 0.953 | |
|  |  | | Pfkl | | Phosphofructokinase | | 0.951 | |
|  |  | | Pgm2 | | Phosphoglucomutase 2 | | 0.95 | |
|  |  | | Pfkfb3 | | 6-phosphofructo-2-kinase/fructose-2,6-biphosphatase 3 | | 0.947 | |
|  |  | | Pfkfb1 | | 6-phosphofructo-2-kinase/fructose-2,6-biphosphatase 1 | | 0.947 | |
|  |  | | Pfkfb2 | | 6-phosphofructo-2-kinase/fructose-2,6-biphosphatase 2 | | 0.947 | |
|  |  | | Pfkfb4 | | 6-phosphofructo-2-kinase/fructose-2,6-biphosphatase 4 | | 0.947 | |
|  |  | |  | |  | |  | |
|  | NME5 | | POLR1B | | Polymerase (RNA) I polypeptide B Gene | | 0.923 | |
|  |  | | POLR3B | | Polymerase (RNA) III (DNA directed) polypeptide B Gene | | 0.914 | |
|  |  | | POLR2B | | Polymerase (RNA) II (DNA directed) polypeptide B Gene | | 0.914 | |
|  |  | | AK7 | | Adenylate kinase 7 Gene | | 0.91 | |
|  |  | | GUCY1A3 | | Guanylate cyclase 1, soluble, alpha 3 Gene | | 0.91 | |
|  |  | | DTYMK | | Midylate kinase Gene | | 0.91 | |
|  |  | | ADCY8 | | Adenylate cyclase 8 Gene | | 0.906 | |
|  |  | | ITPA | | Inosine triphosphatase (nucleoside triphosphate pyrophosphatase) Gene | | 0.904 | |
|  |  | | PKLR | | Pyruvate kinase liver and red blood cell Gene | | 0.904 | |
|  |  | |  | |  | |  | |
|  | AKAP4 | | CABYR | | Calcium-binding tyrosine-(Y)-phosphorylation regulated (fibrousheathin 2) Gene | | 0.943 | |
|  |  | | FSIP2 | | Fibrous sheath-interacting protein 2 Gene | | 0.822 | |
|  |  | | TNP2 | | Transition protein 2 Gene | | 0.792 | |
|  |  | | TNP1 | | Transition protein 1 Gene | | 0.789 | |
|  |  | | LDHC | | Lactate dehydrogenase C Gene | | 0.716 | |
|  |  | | FSIP1 | | Fibrous sheath-interacting protein 1 Gene | | 0.716 | |
|  |  | | ROPN1 | | Ropporin, rhophilin associated protein 1 Gene | | 0.706 | |
|  |  | | LATS1 | | Large tumor suppressor Gene | | 0.665 | |
|  |  | | SPAG6 | | Sperm associated antigen 6 Gene | | 0.658 | |
|  |  | | PGK2 | | Phosphoglycerate kinase 2 Gene | | 0.63 | |
|  |  | |  | |  | |  | |
|  | ARIH1 | | UBE2L6 | | Ubiquitin-conjugating enzyme E2L 6 Gene | | 0.961 | |
|  |  | | EIF4E2 | | Eukaryotic translation initiation factor 4E member 2 Gene | | 0.928 | |
|  |  | | JAK1 | | Janus kinase 1 Gene | | 0.906 | |
|  |  | | PPM1B | | Protein phosphatase 1B, magnesium dependent, beta isoform Gene | | 0.904 | |
|  |  | | UBE2N | | Ubiquitin-conjugating enzyme E2N Gene | | 0.903 | |
|  |  | | UBE2E1 | | Ubiquitin-conjugating enzyme E2E 1, UBC 4/5 homolog (yeast) Gene | | 0.903 | |
|  |  | | IRF3 | | Interferon regulatory factor 3 Gene | | 0.899 | |
|  |  | | IFIT1 | | Interferon-induced protein with tetratricopeptide repeats 1 Gene | | 0.899 | |
|  |  | | PLCG1 | | Phospholipase C , gamma 1 | | 0.899 | |
|  |  | |  | |  | |  | |
|  | RASSF7 | |  | |  | |  | |
|  |  | | RASSF6 | | Ras association (RalGDS/AF-6) domain family member 6 Gene | | 0.77 | |
|  |  | | RASSF1 | | Ras association (RalGDS/AF-6) domain family member 1 Gene | | 0.56 | |
|  |  | | RASSF2 | | Ras association (RalGDS/AF-6) domain family member 2 Gene | | 0.56 | |
|  |  | | RASSF5 | | Ras association (RalGDS/AF-6) domain family member 5 Gene | | 0.52 | |
|  |  | | MOAP1 | | Modulator of apoptosis 1 Gene | | 0.49 | |
| HMGB1 | |  | |  | |  | |  |
|  | | AGER | | Advanced glycosylation end product-specific receptor | | 0.98 | |  |
|  | | HMGB2 | | High mobility group box 2 | | 0.98 | |  |
|  | | NFKB1 | | Nuclear factor of kappa light polypeptide gene enhancer in B cells 1 | | 0.97 | |  |
|  | | RELA | | v-Rel reticuloendotheliosis viral oncogene homolog A | | 0.97 | |  |
|  | | TRP53 | | Transformation related protein 53 | | 0.96 | |  |
|  | | CHUK | | Conserved helix-loop-helix ubiquitous kinase | | 0.96 | |  |
|  | | IKBKB | | Inhibitor of kappaB kinase beta | | 0.96 | |  |
|  | | S100B | | S100 protein, beta polypeptide | | 0.95 | |  |
|  | | NFKB2 | | Nuclear factor of kappa light polypeptide gene enhancer in B cells 2 | | 0.95 | |  |
|  | | TLR4 | | Toll-like receptor 4 | | 0.94 | |  |
|  | | TLR2 | | Toll-like receptor 2 | | 0.93 | |  |
